# ON THE “ACTION” OF DOING HEALTHY AGING AS OPPOSED TO “HAVING” GOOD HEALTH

**DOI:** 10.1093/geroni/igac059.1060

**Published:** 2022-12-20

**Authors:** Jordan Lewis

**Affiliations:** University of Minnesota Medical School, Duluth, Minnesota, United States

## Abstract

The field of successful aging continues to grow and expand as authors investigate this topic in BIPOC populations, broadening the scope of successful aging, including Indigenous communities in the Arctic. Successful aging in Alaska Native communities is still a young, but growing, field of research, and this field of study places Indigenous voices at the forefront. This presentation will present data from 11 years of interviews with 108 Alaska Native Elders from rural and urban communities across the State of Alaska and highlight how successful aging is more of an “action” of doing healthy aging as opposed to the Western notion of “having” good health. The findings of this presentation will discuss how Alaska Native Elders’ understanding, or doing, successful aging can be applied to everyday life, similarities and differences with BIPOC studies on successful aging, and serve as an act of resistance against western notions of successful aging.

